# Supplementary material for: The interactome of intact mitochondria by cross-linking mass spectrometry provides evidence for coexisting respiratory supercomplexes
Source: Mol Cell Proteomics. 2017 Dec 8;17(2):216–32. doi: 10.1074/mcp.RA117.000470 (PMC5795388; doi:10.1074/mcp.RA117.000470)
Supplement: Supplemental Data [file supp_RA117.000470_133922_0_supp_23978_fzffwf.zip › spectra_annotation/mito_spectra_annotation/130-1-3-1-7-1.pdf]

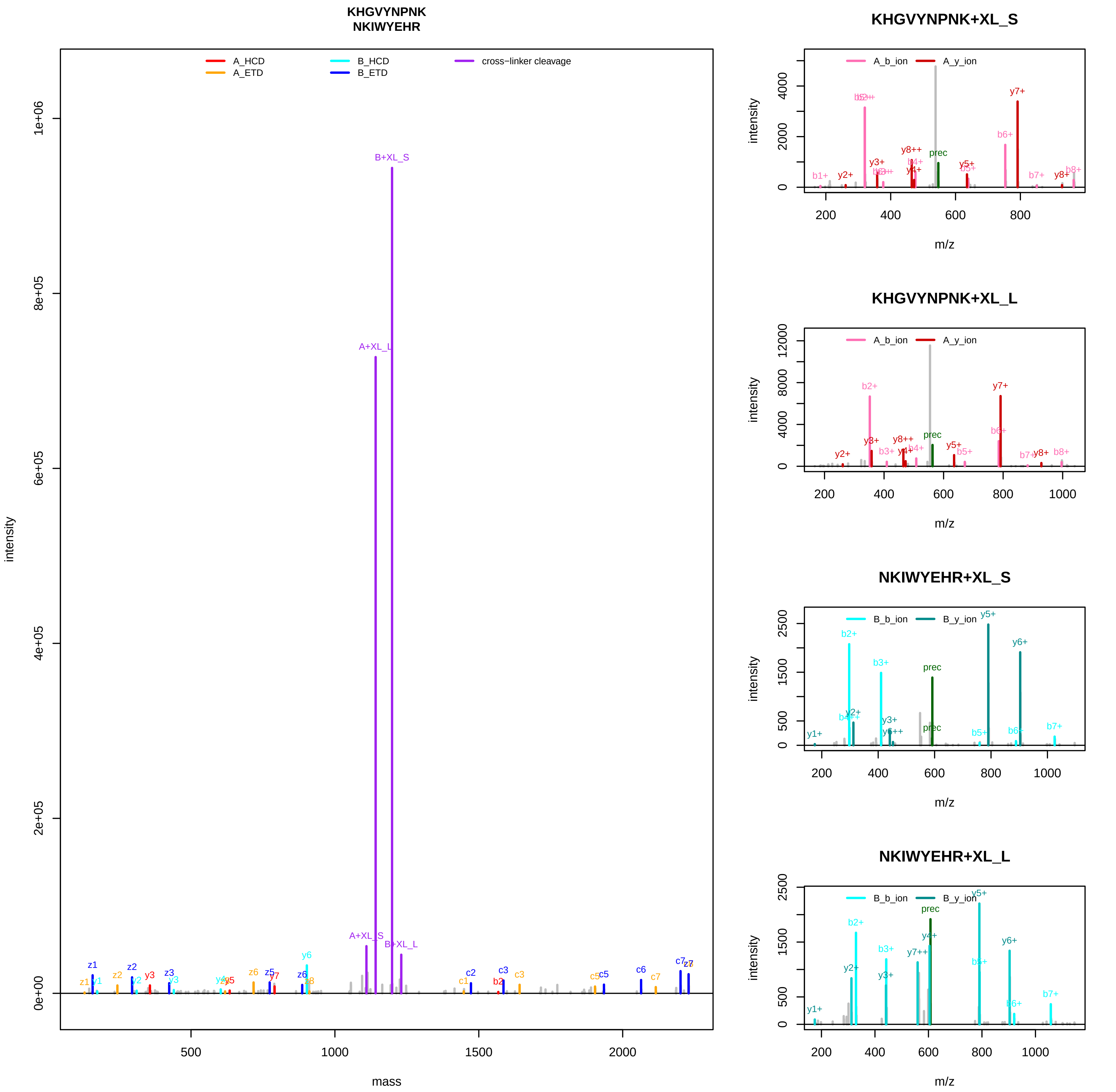
KHGVYNPNK
NKIWYEHR
A_HCD
A_ETD
B_HCD
B_ETD
cross−linker cleavage
intensity
mass
0e+00
2e+05
4e+05
6e+05
8e+05
1e+06
500
1000
1500
2000
B+XL_S
A+XL_L
A+XL_S
B+XL_L
z1
y1
z2
y2
y3
z3
y4
y5
z6
z5
y7
z6
y6
c1
c2
b2
c3
c3
c5
c5
c6
c7
c7
KHGVYNPNK+XL_S
A_b_ion
A_y_ion
m/z
prec
y7+
b6+
y8++
b1+
y2+
y3+
y4+
y5+
b5+
b7+
y8+
b8+
KHGVYNPNK+XL_L
b2+
y3+
b3+
y8++
b4+
b5+
b7+
NKIWYEHR+XL_S
B_b_ion
B_y_ion
y5+
b2+
y6+
b3+
y2+
y3+
y6++
y1+
b5+
b6+
b7+
NKIWYEHR+XL_L
y4+
y7++
y2+
